# Dynamic Expression of Sox2, Gata3, and Prox1 during Primary Auditory Neuron Development in the Mammalian Cochlea

**DOI:** 10.1371/journal.pone.0170568

**Published:** 2017-01-24

**Authors:** Koji Nishimura, Teppei Noda, Alain Dabdoub

**Affiliations:** 1 Shiga Medical Center Research Institute, Moriyama, Shiga, Japan; 2 Biological Sciences, Sunnybrook Research Institute, Toronto, Ontario, Canada; 3 Department of Otolaryngology – Head & Neck Surgery, University of Toronto, Toronto, Ontario, Canada; 4 Department of Laboratory Medicine and Pathobiology, University of Toronto, Toronto, Ontario, Canada; University of Washington, UNITED STATES

## Abstract

Primary auditory neurons (PANs) connect cochlear sensory hair cells in the mammalian inner ear to cochlear nucleus neurons in the brainstem. PANs develop from neuroblasts delaminated from the proneurosensory domain of the otocyst and keep maturing until the onset of hearing after birth. There are two types of PANs: type I, which innervate the inner hair cells (IHCs), and type II, which innervate the outer hair cells (OHCs). Glial cells surrounding these neurons originate from neural crest cells and migrate to the spiral ganglion. Several transcription factors are known to regulate the development and differentiation of PANs. Here we systematically examined the spatiotemporal expression of five transcription factors: Sox2, Sox10, Gata3, Mafb, and Prox1 from early delamination at embryonic day (E) 10.5 to adult. We found that Sox2 and Sox10 were initially expressed in the proneurosensory cells in the otocyst (E10.5). By E12.75 both Sox2 and Sox10 were downregulated in the developing PANs; however, Sox2 expression transiently increased in the neurons around birth. Furthermore, both Sox2 and Sox10 continued to be expressed in spiral ganglion glial cells. We also show that Gata3 and Prox1 were first expressed in all developing neurons, followed by a decrease in expression of Gata3 and Mafb in type I PANs and Prox1 in type II PANs as they matured. Moreover, we describe two subtypes of type II neurons based on Peripherin expression. These results suggest that Sox2, Gata3 and Prox1 play a role during neurogenesis as well as maturation of the PANs.

## Introduction

Primary auditory neurons (PANs), also known as spiral ganglion (SG) neurons, receive chemical signals from cochlear hair cells and transmit the information to the central cochlear nucleus (CN) in the brainstem (see review by Dabdoub and Fritzsch [1]). Neuronal precursors delaminate from the otocyst at the early stage of inner ear development and form PANs [2]. A number of transcription factors that are involved in neurogenesis as well as specification of PANs have been identified: Neurog1 [3, 4], NeuroD1 [5], Gata3 [6, 7] and Sox2 [8–10]. There are two types of PANs. Type I cells comprise 95% of all PANs within the cochlear nerve and have bipolar neurites that connect a single inner hair cell (IHC) with the central CN. Type II PANs make up the remaining 5% of all PANs and have pseudo-unipolar neurites that connect multiple outer hair cells (OHCs) with the CN [11, 12]. The biological differences between type I and type II neurons have been reported [13–21] (also see review on type II PANs by Zhang and Coate [22]). More recently, it was argued that type II PANs should be nociceptors mediating auditory pain and do not drive the olivocochlear reflex [23, 24]; however, it remains unknown how they develop from a common neuroblast.

Concomitant with delamination of neuroblasts, glial cells also migrate from the neural crest [25, 26] and serve as supporting cells for PANs. Myelinating Schwann cells, a glial subtype, surround the peripheral process of type I PANs and non-myelinating Schwann cells surround the peripheral processes of type II PANs. As with glial cells associated with other peripheral nerves, the glia surrounding PANs are important for growth, maintenance and function of the PANs. Supporting glia likely play a role in neuronal pathogenesis [27] as well as provide chemotactic signals for proper pathfinding and innervation of the organ of Corti by afferent PANs [28].

Here we focus on the expression of two groups of transcription factors; the SRY high mobility group box transcription factors Sox2 and Sox10 and another set of transcription factors expressed in embryonic PANs that includes a zinc finger transcription factor Gata3, a leucine-zipper transcription factor Mafb, and a homeobox transcription factor Prox1. Sox2 and Sox10 are first expressed in the developing proneurosensory region of the otocyst and continue to be expressed in glia throughout adulthood [8, 29]; however, changes in expression of these factors during development and maturation of PANs remains unclear. Developing PANs also express Gata3, Mafb and Prox1 until neonatal stages [6, 30–35]; however, the spatial and temporal patterns of gene expression in maturing PANs for these transcription factors have not yet been resolved. To elucidate the changes in expression that may occur during development we performed comprehensive spatiotemporal analyses of these five transcription factors from embryonic to adult stages. This study provides new insights into aspects of gene expression in spiral ganglion development; the involvement of Sox2 in PAN neurogenesis and maturation, Gata3 in type II PAN maturation and Prox1 in type I PAN maturation.

## Materials and Methods

### Animals

Care and euthanasia of CD-1 mice (Charles River laboratory) and *Sox2*^*EGFP/+*^ knock-in mice (Jackson Laboratories, Strain Name: B6;129S-*Sox2tm2Hoch*/J; stock number, 017592) [36] used in this study was approved by the Sunnybrook Research Institute Animal Care Committee, and conformed to IACUC regulations. Mice were maintained in habitat enriched (Bioserv mouse Igloo) isolation cages with automated watering under 12 hour light/12 hour dark cycles at 21°C. Fewer than five adult animals were housed in each cage; cages were changed weekly. Water and food were available ad libitum. Mice were fed the standard irradiated diet and euthanized by CO_2_. We used at least two cochleae from three different litters at each developmental stage.

### Tissue preparation

We bred *Sox2*^*EGFP/+*^ knock-in mice (Sox2-EGFP), in which the *Sox2* open reading frame is replaced by *EGFP* [36], with CD-1 female mice as *Sox2*^*EGFP/EGFP*^ are embryonic lethal [37]. We harvested mouse tissue at embryonic day (E) 10.5, 12.75, 17.5, from Sox2-EGFP mice, and E10.5, E12.75, E13.5, E15.5, E17.5, postnatal day (P) 0, P1, P5, P6, P14, P30, P35 and P40 from CD-1 mice. After dissecting the temporal bone and removing surrounding tissue in cold phosphate buffer saline (PBS), cochleae were fixed for 30 min at RT in 2% paraformaldehyde (for Peripherin), 2 hours (for Sox2) or overnight (for other antibodies) in 4% paraformaldehyde at room temperature. P5 or older cochleae were decalcified with 5% EDTA in PBS for up to 5 days at room temperature. Then, the cochleae were immersed in 30% sucrose in PBS overnight and embedded into OCT compound (TissueTek), and cryosectioned with 10 μm thickness. Specimens were mounted on MAS-GP coated slide glass (Matsunami).

### Immunohistochemistry

Immunohistochemistry was performed as described previously [38]. For cochlear immunohistochemistry the primary antibodies used are in Table 1. Notably, we used TuJ1 antibodies to label neuron specific class III β tubulin [39]. We used Alexa Fluor 488 or 546 labeled donkey anti-goat IgG, Alexa Flour 546 and 647 labeled anti-rabbit IgG, and Alexa Fluor 488 and 647 labeled donkey anti-mouse IgG as secondary antibodies. Nuclei were stained with 4',6-diamidino-2-phenylindole (DAPI).

**Table 1:** List of primary antibodies. All antibodies were diluted in 10% donkey serum and 0.5% Triton X-100 in Tris-buffered saline (TBS) at the concentrations indicated in the table.

Antibody	Antigen	Host	Supplier	Catalog #	Dilution
Sox2	A peptide mapping near the C-terminus of Sox2 of human origin	Goat polyclonal	Santa Cruz	Y-17	1:250
Sox10	A peptide mapping near the N-terminus of Sox10 of human origin	Goat polyclonal	Santa Cruz	N-20	1:250
GATA3	*E*. *coli*-derived recombinant human GATA3	Goat polyclonal	R&D systems	AF2605	1:250
Prox1	*E*. *coli*-derived recombinant human Prox1	Goat polyclonal	R&D Systems	AF2727	1:500
Mafb	Transcription factor Mafb recombinant protein epitope signature tag (PrEST)	Rabbit polyclonal	Sigma-Aldrich	HPA005653	1:200
TuJ1	microtubules derived from rat brain	Mouse monoclonal	Covance	MMS-435P	1:1000
TuJ1	a synthetic peptide corresponding to amino acid residues 441–450 of human β-tubulin III (Ala 446 to Ser446 substitution) with N-terminal added cysteine, conjugated to KLH	Rabbit polyclonal	Sigma-Aldrich	T2200	1:500
NF200	The C-terminal tail segment of enzymatically dephosphorylated pig Neurofilament H-subunit	Mouse monoclonal	Sigma-Aldrich	N0142	1:250
Peripherin	Electrophoretically pure trp-E-Peripherin fusion protein, containing all but the 4 N terminal amino acids of rat Peripherin	Rabbit polyclonal	Millipore	AB1530	1:5000

## Results

### Sox2 expression in the spiral ganglion during mouse cochlear development

**Fig 1:**
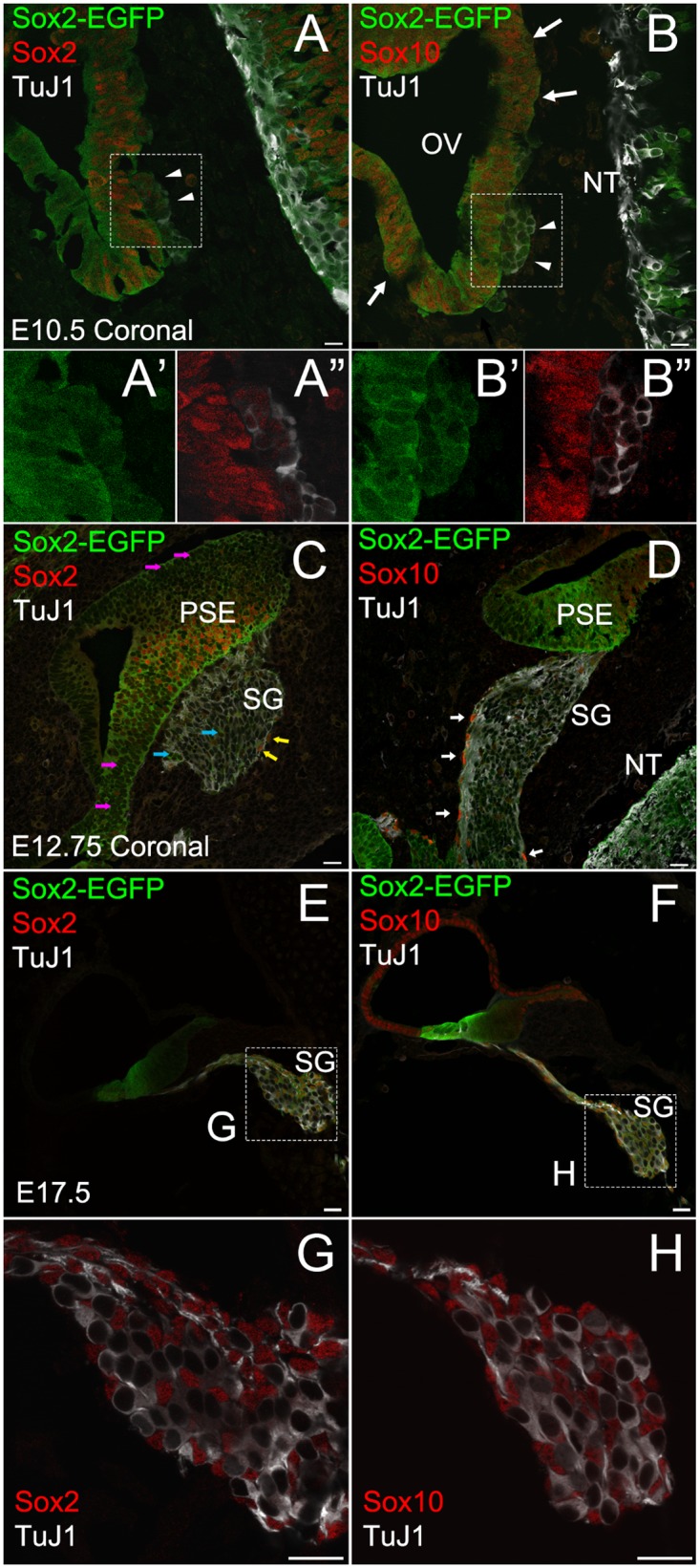
Sox2 expression in the spiral ganglion of Sox2-EGFP mice during cochlear development. **A:** A coronal cross section through the otic vesicle (OV) from E10.5 Sox2-EGFP mouse immunostained against Sox2 (red) and TuJ1 (white). Arrowheads indicate delaminating neuroblasts. TuJ1 is expressed in delaminating neuroblasts and cells in the neural tube (NT). Sox2-EGFP (green) overlaps Sox2 protein. **A’**: High magnification image of A with Sox2-EGFP channel only. **A”**: The same image as in A’ with Sox2 and TuJ1. **B:** A coronal cross section through the otic vesicle (OV) from E10.5 Sox2-EGFP mouse immunostained against Sox10 (red) and TuJ1 (white). Cells in OV express Sox10 (arrows) but Sox10 is downregulated in neuroblasts (arrowheads). **B’:** High magnification image of B with Sox2-EGFP channel only. **B”:** The same image as in B’ with Sox10 and TuJ1. **C:** A coronal section through OV from E12.75 Sox2-EGFP mouse immunostained against Sox2 (red) and TuJ1 (white) showing developing spiral ganglion (SG) and prosensory epithelium (PSE). **D:** A coronal section through OV from E12.75 Sox2-GFP mouse immunostained against Sox10 (red) and TuJ1 (white). Sox10^+^ glial cells enwrap SG. **E:** A cochlear cross section from E17.5 Sox2-EGFP mouse immunostained against Sox2 (red) and TuJ1 (white). **F:** A cochlear section from E17.5 Sox2-EGFP mouse immunostained against Sox10 (red) and TuJ1 (white). **G:** High magnification image of **E**, except for Sox2-EGFP. **H:** High magnification image of **F**, except for Sox2-EGFP. Blue arrows in **C** indicate Sox2-EGFP^+^/Sox2^-^ cells in SG. Yellow arrows in **C** indicate Sox2-EGFP^+^/Sox2^+^ cells in SG. Pink arrows in **C** indicate Sox2-EGFP^+^/Sox2^-^ cells in the otocyst. White arrows in **D** indicate Sox2-EGFP^+^/Sox10^+^ cells. Scale bars: 20 μm.

**Fig 2:**
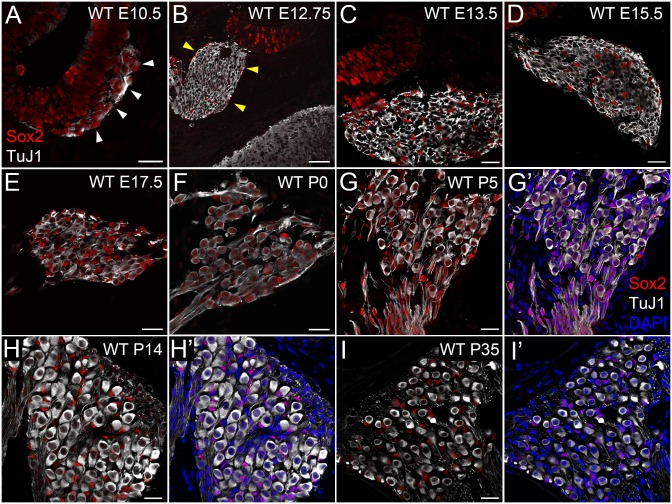
Immunostaining of wild type mice reveals dynamic Sox2 expression in developing primary auditory neurons. Immunohistochemistry for Sox2 (red) and TuJ1 (white). **A:** A cross section through the ventral otocyst from E10.5 CD-1 embryos. White arrowheads indicate delaminating neuroblasts expressing Sox2. **B:** A coronal section through the cochlea from E12.75 CD-1 embryos. Yellow arrowheads indicate Sox2 positive cells. These cells were negative for TuJ1. **C:** A cochlear cross section from E13.5 CD-1 mice. Sox2 (red) was strongly detected in TuJ1-negative glial cells, but not detected in TuJ1-positive cells. **D:** A cochlear cross section from E15.5 CD-1 mice. Sox2 (red) was strongly detected in TuJ1-negative glial cells, but not detected in TuJ1-positive cells. **E:** A cochlear cross section from E17.5 CD-1 embryos. Sox2 (red) was strongly detected in TuJ1-negative glial cells, and weakly detected in TuJ1-positive cells. **F:** A cochlear cross section from P0 CD-1 mice. Sox2 (red) was strongly detected in TuJ1-negative cells, and moderately detected in TuJ1-positive cells. **G:** A cochlear cross section from P5 CD-1 mice. Sox2 (red) was strongly detected in TuJ1-negative cells, and moderately detected in TuJ1-positive cells. **G’: G** with DAPI channel. Non-neural, non-glial cells neither expressed Sox2 nor TuJ1. **H:** A cochlear cross section from P14 CD-1 mice. Sox2 (red) in TuJ1 positive cells was no longer detected. **H’: H** with DAPI channel. **I:** A cochlear cross section from P35 CD-1 mice. Sox2 (red) in TuJ1 positive cells was not detected. **I’: I** with DAPI channel. Scale bar in **B**: 50 μm; others: 20 μm.

Cells in the spiral ganglion (SG) include PANs, non-neural cells composed of glial cells that surround PANs and mesenchymal cells such as fibroblasts [40]. We investigated which cell type(s) in the spiral ganglion express Sox2 at early developmental stages by performing immunohistochemistry on cochlear sections from *Sox2*^*EGFP/+*^ mice (Sox2-EGFP). Since Sox10 labels neural crest derived cells including Schwann cells in the cochleovestibular ganglion (CVG) [28, 40, 41], we used Sox10 as a marker for glial cells in the spiral ganglion. We used TuJ1 as a marker for delaminating neuroblasts as well as delaminated neurons [29, 42]. At E10.5, when neuroblasts are in the process of delaminating from the anteroventral otic vesicle [4], TuJ1 positive delaminating neuroblasts as well as cells in the neural tube and in the otocyst expressed Sox2-EGFP and Sox2 protein (Fig 1A arrowheads, Fig 1A’ and 1A”). Sox10 positive cells were present in the otocyst (Fig 1B arrows) in accordance with previous reports [29, 41]. Sox2-EGFP^+^ neuroblasts were negative for Sox10 (Fig 1B arrowheads, Fig 1B’ and 1B”), indicating neuroblasts downregulated Sox10 after they delaminated from the otocyst. By E12.75, neuroblasts have already delaminated from the otocyst and formed the spiral ganglion, and neural crest cells form corridors delineating the path of migratory neuroblasts [25, 43]. Our data also showed Sox10^+^ neural crest cells were enwrapping the SG (Fig 1D, indicated by white arrows). Enwrapping neural crest cells as well as cells in the SG expressed Sox2-EGFP (Fig 1C and 1D). Enwrapping neural crest cells but not TuJ1 positive neuronal cells in the SG expressed Sox2 protein (Fig 1C). At this stage we observed that while neurons did not express Sox2 protein, Sox2-EGFP was present at E12.75 (Fig 1C, blue arrows) likely due to the longer half-life of EGFP [36]. Moreover, we also observed cells in the otocyst that are positive for Sox2-EGFP but negative for Sox2 protein (Fig 1C, pink arrows), agreeing with a previous report showing that Sox2-expressing cells in the early otocyst give rise to large numbers of Sox2 negative cochlear non-sensory epithelial cells [10]. Altogether our data indicate that migrating glial cells expressed Sox2 but post-mitotic neurons in the CVG downregulated Sox2 (blue arrows in Fig 1C represent Sox2-EGFP^+^/Sox2^-^ neurons, and yellow arrows represent Sox2-EGFP^+^/Sox2^+^ glial cells. Pink arrows in Fig 1C represent Sox2-EGFP^+^/Sox2^-^ cochlear non-sensory epithelial cells). At E17.5, neural crest glial cells were integrated throughout the spiral ganglion [25]. Neither Sox2 nor Sox10 antibodies labeled TuJ1^+^ neurons at this stage (Fig 1E–1H). Sox2-EGFP was colocalized with Sox10^+^ glial cells (Fig 1F) and Sox2 immunolabelling (Fig 1E), indicating that Sox10^+^ glial cells also expressed Sox2. Considering Sox2 expression levels in Sox2-EGFP mice is about half that of wild type mice [44, 45] and neurons at E12.75 and E17.5 did not express Sox2 protein, we examined Sox2 expression in PANs of wild type mice. Sox2 localization of PANs in WT mice at E10.5 and E12.75 was consistent with that in Sox2-EGFP mice (Fig 2A and 2B). Not neurons but glial cells in WT expressed Sox2 at E13.5 (Fig 2C) and E15.5 (Fig 2D); however, at E17.5, P0 and P5, TuJ1^+^ neurons of WT mice expressed Sox2 protein (Fig 2E–2G’). Having looked at E17.5 (Fig 2E), P0 (Fig 2F) and P5 (Fig 2G and 2G’), we found Sox2 expression level in PANs have a second peak around P0, indicating Sox2 was transiently expressed in PANs around birth. Furthermore, PANs of WT mice at P14 and P35 downregulated Sox2 (Fig 2H–2I’) and Sox2 expression was maintained only in TuJ1-negative non-neural cells surrounding PANs—glial cells.

### Gata3 localization during development of the spiral ganglion

**Fig 3:**
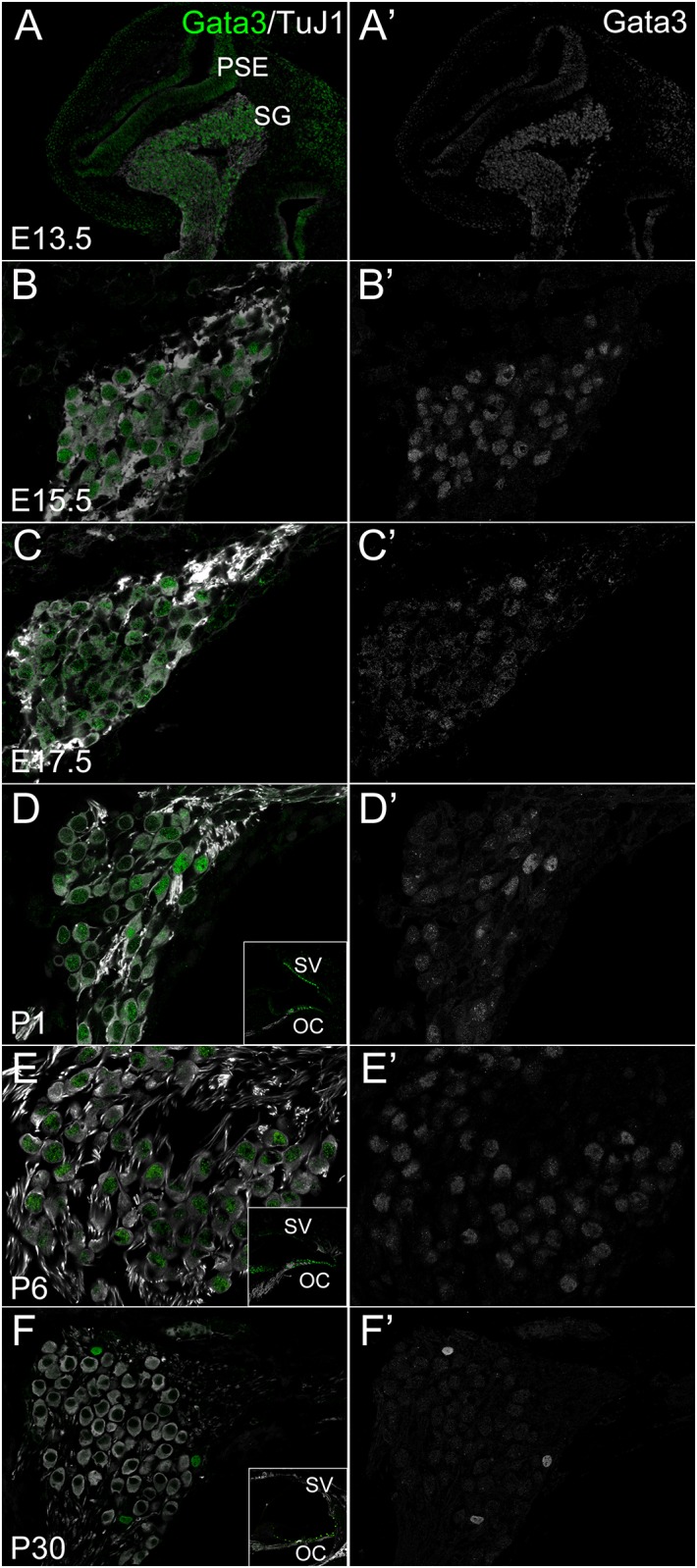
Spatiotemporal expression of Gata3 in auditory neurons. Cochlear cross sections (WT) from E13.5 to P30 immunostained against Gata3 and TuJ1. Gata3 (green) and TuJ1 (white) (**A**−**F**) and single channel of Gata3 (white) (**A’**−**F’**). **A:** At E13.5, cells in SG (spiral ganglion) and PSE (prosensory epithelium) expressed Gata3. **B:** At E15.5, Gata3 was positive in the nuclei of all the PANs. At E17.5 (**C**) and P1 (**D**), Gata3 was positive in the nuclei of all the PANs, with expression gradient. Yellow arrowheads in **C**−**D’** indicate strong localization of Gata3 to the nuclei. **E:** At P6, PANs continued to express Gata3. **F:** At P30, there are clearly two different types of cells: a couple of nuclear-localized Gata3 expressing cells and the other Gata3-negative cells. Insets of **D**−**F** shows Gata3 (green) and TuJ1 (white) expression at the organ of Corti and the stria vascularis. SG: spiral ganglion, PSE: prosensory epithelium, SV: stria vascularis, OC: organ of Corti. Scale bars in **A’**, 100 μm; **B’**−**F’**, 20 μm.

**Fig 4:**
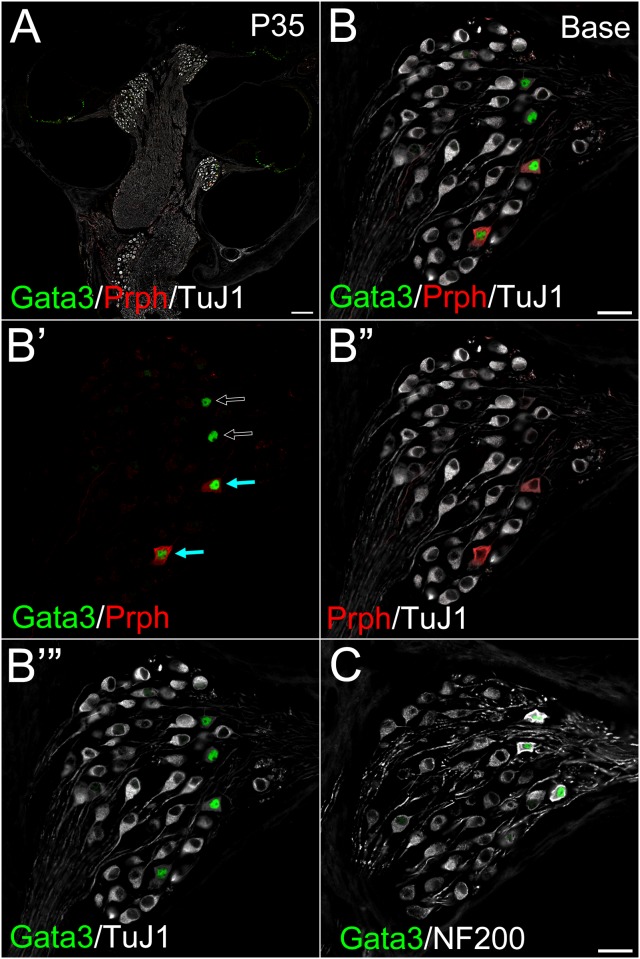
Localization of Peripherin and Gata3 at P35 in the spiral ganglion. A cochlear cross section of postnatal SG (WT) at P35 immunostained against Gata3 (green), Peripherin (red) and TuJ1 (white) (**A**−**B’”**), or Gata3 (green) and NF200 (white) (**C**). Some Gata3 expressing cells were Peripherin positive, which weakly expressed TuJ1 (indicated by blue arrows in **B’**). Some Gata3 expressing cells were negative for Peripherin, which weakly expressed TuJ1 (indicated by open arrows in **B’**). (**A**−**B’”**). **A:** Low magnification view of a cochlear cross section from WT P35 mouse. Gata3 (green) was localized in the nuclei of a few SG cells. Gata3 was also positive in the cells of the organ of Corti including hair cells and supporting cells. **B:** High magnification view of **A**, focused on basal SG. **B’:** The same image as in **A**, except for TuJ1. **B”:** the same image as in **A**, except for Gata3. **B’”:** The same image as in **A**, except for Peripherin. **C:** Another cochlear cross section from P35 mouse. Gata3 (green) strongly positive cells also strongly expressed NF200 (white). Scale bar in **A**, 100 μm. Scale bars in **B** and **C**, 20 μm.

**Fig 5:**
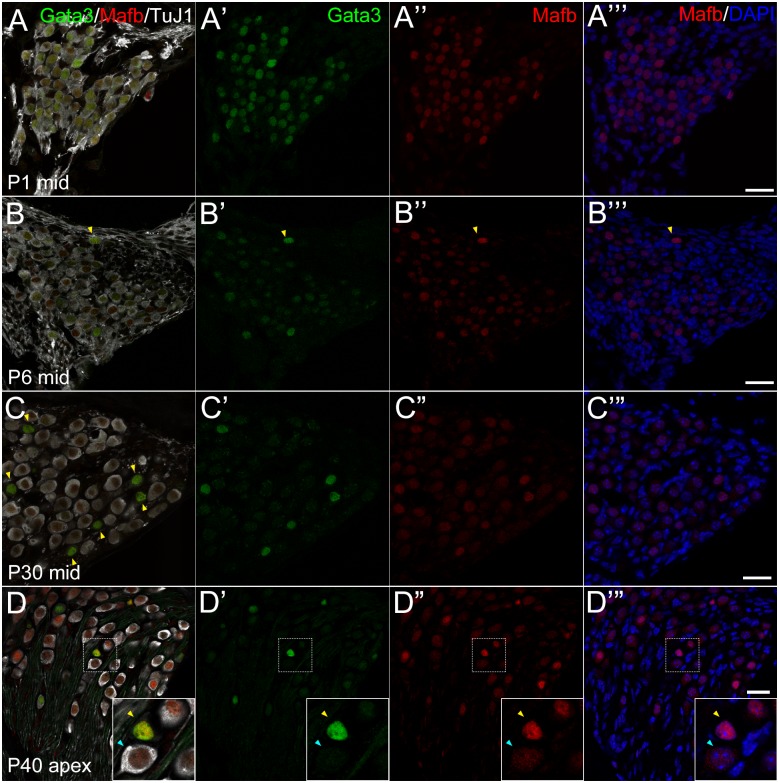
Localization of Mafb and Gata3 at postnatal stages. Cochlear cross sections of postnatal SG at P1 (**A**), P6 (**B**), P30 (**C**) and P40 (**D**) immunostained against TuJ1 (white), Gata3 (green) and Mafb (red). Each row shows different stages. **A**−**A”‘:** At P1, Mafb (red) was co-localized in the nuclei with Gata3 (green) in TuJ1 (white)-positive PANs. **B**−**B”‘:** At P6, like P1, Mafb was localized in most of PANs, and strongly detected in Gata3 strongly positive cells (indicated by yellow arrowheads). **C**−**C”‘:** At P30, Mafb was co-localized with Gata3 expression like P6. TuJ1 was negative or weakly positive in Gata3 and Mafb double positive cells indicated by yellow arrowheads in **C**. **D**−**D”‘:** At P40, Mafb expression was the same as P30. High magnification view is shown in the inset, indicating there existed two types of Mafb-positive cells: Gata3 and Mafb double positive cells, which have rather small cell nucleus and did not express TuJ1 (yellow arrowhead); and Gata3 negative, Mafb cytoplasmic positive, and TuJ1 positive cells, which have larger cell body (blue arrowhead). Nuclei were stained with DAPI (blue). Scale bars, 20 μm.

We studied the expression of another transcription factor Gata3 in the developing and mature inner ear. The spatiotemporal expression of Gata3 has been reported in embryonic and early postnatal PANs [6, 30, 33, 46, 47], but not in older PANs, except for an expression study using microarrays [32]. For a comprehensive review on Gata3 in PAN development, see review chapter by Goodrich [1]. Moreover, early deletion of Gata3 results in complete neurosensory ablation of the organ of Corti and PANs [31] whereas delayed ablation of Gata3 results in variable pathfinding errors of PANs [33]. Hence, we investigated whether Gata3 might have a role not only in development but also in maturation. To determine its role in maintenance and maturation more thoroughly, as a first step we performed immunohistochemical staining for Gata3 and co-stained with TuJ1 at various developmental stages (Fig 3). Agreeing with previous reports of Gata3 localization at embryonic stages, Gata3 protein was detected in the nuclei of developing PANs and the cochlear epithelia (Fig 3A–3C). At postnatal stages (Fig 3D–3F), there were two different subsets of PANs regarding expression level of nuclear-localized Gata3. This change of Gata3 immunofluorescence among different PANs started as early as E17.5 (Fig 3C), and was maintained at least until P40 (Figs 3D–3F, 4 and 5D). During this period, a subset of neurons maintained nuclear Gata3 expression (indicated by arrowheads in Fig 3C’–3E’), whereas the remaining neurons downregulated Gata3. As neuronal maturation progressed, this difference became larger, resulting in very weak signal detection in most PANs at P30 (Figs 3F and 5C), P35 (Fig 4 and S2 Fig) and at P40 (Fig 5D). Interestingly, in cells that strongly expressed Gata3, a pan-neuronal marker, TuJ1 signal was detected until P6 (Fig 3E). In contrast, from P14 onwards, cells that strongly expressed Gata3 had weak or no TuJ1 expression (Figs 3F, 4 and 5C–5D, S1 and S2 Figs). These data suggest that there are at least two different cell populations in PANs discriminated by Gata3 expression.

### Gata3 marks type II neurons at adult stages

Peripherin, the type III intermediate neurofilament, marks mature type II PANs [15, 16]. Given that TuJ1 is negative in Peripherin positive type II PANs at later stages in various species [13, 18, 19, 48] and our data showing that Gata3 and TuJ1 expression was mutually exclusive in older PANs, we postulated that Gata3 marks nuclei of type II PANs. To investigate this, we performed immunostaining for Gata3, TuJ1 and Peripherin at P14, when type II PANs have been already pruned [13]. Peripherin positive cells did not express TuJ1 and strongly expressed Gata3 (S1 Fig), suggesting Gata3 marks type II PANs. In addition, we confirmed Gata3 and Peripherin double-positive cells are neurons by immunostaining for another neurofilament protein, NF200 (S1 Fig), as previously reported [16]. In addition, Peripherin positive cells were absent at the most apical turn [13], agreeing with our data that very few Peripherin positive cells were detected at the apical turn at P14 (S1F, S1I, S1O, S1R, S1F’, S1I’, S1O’ and S1R’ Fig). At P35, Gata3 positive cells had weak or no TuJ1 expression and strong expression of NF200 (Fig 4A–4C, S2 Fig), suggesting they were type II PANs. Unexpectedly some Gata3 expressing cells with weak TuJ1 and strong NF200 expression were Peripherin negative (indicated by open arrows in Fig 4B’ and S2 Fig) suggesting that there are two classes of type II neurons.

We also tested the localization of a Gata3 downstream transcription factor, Mafb. *Mafb* was first described as a gene related to musculoaponeurotic fibrosarcoma, and now is known to be the responsible gene for the *Kreisler* mutant (*kr/kr*) and Duane syndrome, characterized by inner ear defects and segmentation abnormalities in rhombomeres during early embryogenesis [49, 50]. Yu et al. indicated that Mafb works downstream of Gata3 and contributed to synaptogenesis between IHCs and type I PANs [51]. Here we examined expression of Gata3, which is upstream of Mafb, and localization of Mafb in the postnatal PANs. As previously described [51], Mafb protein was localized in the nuclei of PANs at P1 and P6 (Fig 5A and 5B). At P30 and P40, while Mafb expression has been reported to be translocated to the cytoplasm of most PANs by P15, our data suggested that its nuclear localization is maintained in a small number of cells, especially in Gata3 positive cells (Fig 5C and 5D, yellow arrowhead in the inset). Cells with nuclear expression of Gata3 and Mafb did not express TuJ1, suggesting they were type II PANs.

### Prox1 marks type I primary auditory neurons at juvenile and adult stages

**Fig 6:**
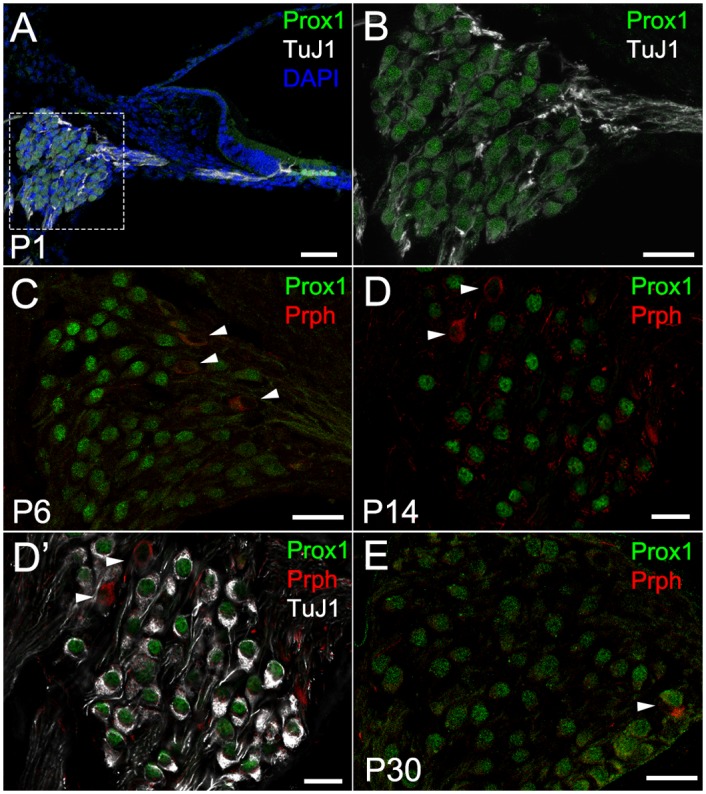
Expression of Prox1 in postnatal PANs. Cochlear cross sections of postnatal SG at P1 (**A**, **B**), P6 (**C**), P14 (**D**, **D’**) and P30 (**E**). **A:** At P1, Prox1 (green) was positive in TuJ1 (white) positive PANs in SG and the supporting cells in the organ of Corti. **B:** High magnification image of SG in **A**, except for DAPI (blue). **C:** Immunostaining for Prox1 (green) and Peripherin (red) at P6. **D:** Immunostaining for Prox1 (green) and Peripherin (red) at P14. **D’:** The same image of **D**, co-stained for TuJ1. **E:** Immunostaining for Prox1 (green) and Peripherin (red) at P30. Arrowheads in **C**, **D**, **D’** and **E** indicate Peripherin-positive but Prox1-negative cells. Scale bars in **A**: 50 μm, in **B**, **C**, **C’**, **D**, **E**: 20 μm.

We also investigated the expression of another transcription factor, Prox1, which is expressed in embryonic and neonatal [34, 35] PANs. Prox1 has been suggested to affect type II PANs’ wiring [34]. We sought to determine its exact spatiotemporal expression in PANs. At P1, when Gata3 and TuJ1 mark all PANs, Prox1 was detected in all TuJ1 positive cells (Fig 6A and 6B). At juvenile and adult stages however, only type I PANs, which were positive for TuJ1 and negative for Peripherin, continued to express Prox1 (Fig 6C–6E, S3 Fig).

## Discussion

Sox2, which belongs to the high-mobility group (HMG) box transcription factors, is unique as it is the only transcription factor that is expressed in embryonic and adult stem cells as well as progenitor cells [36]. Furthermore, adult stem cells expressing Sox2 originate from Sox2 expressing progenitor cells indicating that Sox2 initially plays a role in the development of progenitor cells and continues to be expressed in derivative adult tissues where it indicates stem cells [36]. With regard to inner ear development, Sox2 is the earliest definitive marker of the prosensory domain [52] and remains expressed in the supporting cells and cells in the spiral ganglion at postnatal stages [8, 53–56]. We examined spatiotemporal expression of Sox2 and Sox10 in the developing cochlea focusing on cells in the spiral ganglion. Our results demonstrated Sox2 expression levels had two peaks at the delamination of neuroblasts from otocyst and around birth, in general agreement with the previously reported expression patterns of Sox2 in developing avian [56] and mouse [8] inner ear, and with lineage tracing studies of Sox2 [10]. In summary, Sox2 was first expressed in delaminating neuroblasts and its downregulation is necessary for progression of neurogenesis by Neurogenin1 and NeuroD1 [57]. Sox2 expression was later upregulated in PANs around birth and finally declined to undetectable levels by adult stages. Antagonistic interactions between Sox2 and bHLH transcription factors have been well documented [53, 57, 58], and might explain the upregulation of Sox2 in PANs around birth. For example, the transient downregulation of Neurog1 around birth [32] (http://goodrich.med.harvard.edu/microarray-data.html) could allow for the upregulation in Sox2 expression we observed.

Our results also showed that Sox10 is downregulated in delaminating neuroblasts while another study demonstrated it is still expressed in delaminating neuroblasts [29]. The discrepancy likely comes from the way Sox10 was labeled: we used Sox10 immunohistochemistry to label currently expressed protein while Wakaoka et al. used Sox10-IRES-Venus reporter mice, where the reporter likely has a longer half-life than the Sox10 protein. We can explain the discrepancy between Sox2-EGFP and immunohistological labelling of Sox2 protein in *Sox2*^*EGFP/+*^ knock-in mice in the same manner thus Sox2-EGFP does not always accurately report Sox2 protein expression. Based on our results, Sox10 is downregulated earlier than Sox2 in neuroblasts during delamination.

We also demonstrated spatiotemporal Gata3 expression. It was first expressed in all PANs throughout embryonic development but then downregulated in TuJ1 positive type I PANs as development progressed after birth, being compatible with previous microarray data demonstrating Gata3 expression in PANs peaked at E16 [32]. Interestingly, nuclear Gata3 expression was highly maintained only in a small number of cells also positive for Peripherin at P14 and older stages suggesting that Gata3 labeled postnatal type II neurons. We thus hypothesize that sufficient Gata3 expression is important for diversification and maturation of type II PANs. Since haploinsufficiency of Gata3 causes HDR syndrome [59, 60], characterized by moderate to severe hearing impairment with loss of otoacoustic emission (OAE) [61, 62], the expression level of Gata3 is likely important in the development of the OHC. It is of great interest whether patients with HDR syndrome have abnormalities in type II PANs, but we do not have a clinical test to evaluate this except for temporal bone histopathology. We also demonstrated Prox1 expression, which was detected in all PANs at P1, but only in TuJ1 positive, Peripherin negative type I PANs at older stages.

Our data showed that Mafb nuclear expression was strongly maintained in mature type II PANs, indicating that a Gata3-Mafb transcriptional network, which plays an important role in synaptogenesis between IHCs and type I PANs [51], was also expressed in mature type II PANs at higher levels. To test whether a Gata3-Mafb transcriptional network might have a role in synaptogenesis of type II PANs, which have more synaptic targets than type I PAN do, we need to check whether Mafb mutant mice have any abnormalities in synapses between OHCs and type II PANs.

We observed very few Peripherin positive cells at the apex, agreeing with a previous report [13]. Since type II neurons, which had darkly stained nuclei and a smaller cell body, were distributed throughout the entire cochlear length [63], it is likely that these were type II neurons even though they did not express Peripherin. Cells at the apical turn with strong Gata3 expression should be type II neurons since they also strongly expressed NF200 and weakly expressed TuJ1, which is another characteristic of type II neurons [18, 64, 65]. More recently, it was demonstrated that some tyrosine hydroxylase positive neurons with type II morphology did not express Peripherin [21] and type II PANs existed in Peripherin KO mice [23], both of which support our finding that some type II PANs were Peripherin negative.

**Fig 7:**
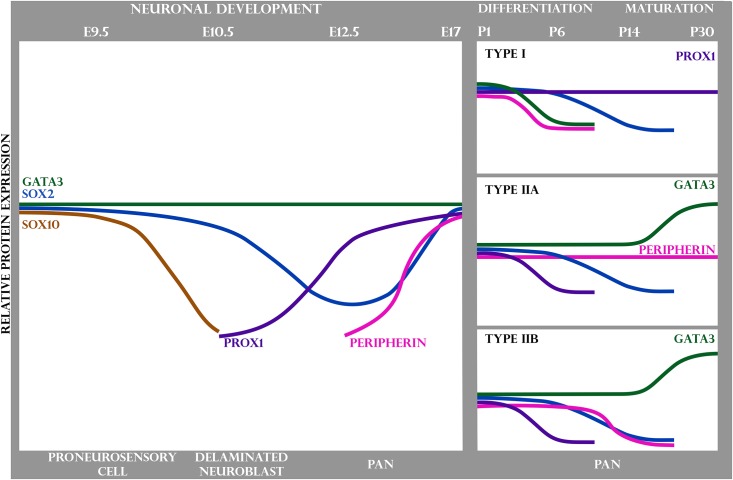
Expression of Sox2, Sox10, Gata3, Prox1 and Peripherin during primary auditory neuron development and maturation. This schematic illustrates the expression of Gata3 (green), Sox2 (blue), Sox10 (brown), Prox1 (purple) and Peripherin (pink) as cells transition from the proneurosensory stage in the developing embryo, to the maturation of PANs in the spiral ganglion. Downward and upward inflections indicate the corresponding decrease and increase in expression, respectively. At E10.5, Sox10 was downregulated as neuroblasts delaminated from the otocyst. At E12.5, Sox2 expression was similarly downregulated and Prox1 began to be expressed in these cells. At E17, Peripherin started to become expressed. At P1, Sox2 was upregulated and maturing PANs began to differ in gene expression as they differentiated into their respective subtypes. All PANs expressed NF200, but only type I PANs strongly expressed TuJ1. Type I PANs downregulated Gata3 early and subsequently downregulated Sox2; however, Prox1 continued to be expressed. Type IIA PANs continued to express Gata3 strongly in adulthood and maintained expression of Peripherin while downregulating both Prox1 and Sox2. Type IIB PANs had a similar expression pattern to type IIA PANs but diverged when they started to downregulate Peripherin.

We summarize our expression data in PANs throughout development and maturation in Fig 7. Our results indicated that Sox2 and Gata3 were initially expressed in the developing spiral ganglion and then Sox2 expression was restricted to glial cells and Gata3 expression was maintained in type II neurons while Prox1 was maintained in type I neurons. While all type II PANs upregulated Gata3, some type II PANs were positive for Peripherin and others were negative, which we define as type IIA PANs and type IIB PANs, respectively.

## Supporting Information

S1 Fig Localization of Peripherin, TuJ1, NF200 and Gata3 at P14 in different cochlear regions. A cochlear cross section of postnatal SG (WT) at P14 immunostained against Gata3 (green), Peripherin (red) and TuJ1 (white). Each column represents different tonotopic positions: the basal turn **(A, D, G, J, M, P)**, the middle turn **(B, E, H, K, N, Q)** and the apical turn **(C, F, I, L, O, R)**. **(A-C)** Gata3 single channel. **(D-F)** Peripherin single channel. **(G-I)** Gata3 and Peripherin double channels. **(J-L)** Gata3 and TuJ1 double channels. **(M-O)** Peripherin and TuJ1 double channels. **(P-R)** Gata3, Peripherin and TuJ1 triple channels. Gata3 expressing cells were positive for Peripherin, but negative for TuJ1 at the basal and middle turn. There was one Peripherin positive cell in the apex, which was negative for TuJ1 (indicated by arrows in **C, F, I, L, O, R**). Scale bar: 50 μm. A cochlear cross section of postnatal SG (WT) at P14 immunostained against Gata3 (green), Peripherin (red) and NF200 (white). Each column represents different tonotopic positions: the basal turn **(A’, D’, G’, J’, M’, P’)**, the middle turn **(B’, E’, H’, K’, N’, Q’)** and the apical turn **(C’, F’, I’, L’, O’, R’)**. **(A’-C’)** Gata3 single channel. **(D’-F’)** Peripherin single channel. **(G’-I’)** Gata3 and Peripherin double channels. **(J’-L’)** Gata3 and NF200 double channels. **(M’-O’)** Peripherin and NF200 double channels. **(P’-R’)** Gata3, Peripherin and NF200 triple channels. Gata3 expressing cells were positive for Peripherin and strongly positive for NF200 at the basal and middle turn. There were no Peripherin positive cells in the apex. Scale bar: 50 μm.
(TIF)

S2 Fig Localization of Peripherin, TuJ1, NF200 and Gata3 at P35 in different cochlear regions. A cochlear cross section of postnatal SG (WT) at P35 immunostained against Gata3 (green), Peripherin (red) and TuJ1 (white). Each column represents different tonotopic positions: the basal turn **(A, D, G, J, M, P)**, the middle turn **(B, E, H, K, N, Q)** and the apical turn **(C, F, I, L, O, R)**. **(A-C)** Gata3 single channel. **(D-F)** Peripherin single channel. **(G-I)** Gata3 and Peripherin double channels. **(J-L)** Gata3 and TuJ1 double channels. **(M-O)** Peripherin and TuJ1 double channels. **(P-R)** Gata3, Peripherin and TuJ1 triple channels. Some Gata3 expressing cells were Peripherin positive, which were TuJ1 negative (indicated by blue arrows). Some Gata3 expressing cells were negative for Peripherin, which were TuJ1 negative (indicated by open arrows). There were no Peripherin positive cells in the apex. Scale bar: 50 μm. A cochlear cross section of postnatal SG (WT) at P35 immunostained against Gata3 (green), Peripherin (red) and NF200 (white). Each column represents different tonotopic positions: the basal turn **(A’, D’, G’, J’, M’, P’)**, the middle turn **(B’, E’, H’, K’, N’, Q’)** and the apical turn **(C’, F’, I’, L’, O’, R’)**. **(A’-C’)** Gata3 single channel. **(D’-F’)** Peripherin single channel. **(G’-I’)** Gata3 and Peripherin double channels. **(J’-L’)** Gata3 and NF200 double channels. **(M’-O’)** Peripherin and NF200 double channels. **(P’-R’)** Gata3, Peripherin and NF200 triple channels. Some Gata3 expressing cells were Peripherin positive, which were NF200 strongly positive (indicated by blue arrows). Some Gata3 expressing cells were negative for Peripherin, which were NF200 strongly positive (indicated by open arrows). There were no Peripherin positive cells in the apex. Scale bar: 50 μm.
(TIF)

S3 Fig Localization of Peripherin, TuJ1, NF200 and Prox1 at P14 in different cochlear region. A cochlear cross section of postnatal SG (WT) at P14 immunostained against Prox1 (green), peripherin (red) and TuJ1 (white). Each column represents different tonotopic positions: the basal turn **(A, D, G, J, M, P)**, the middle turn **(B, E, H, K, N, Q)** and the apical turn **(C, F, I, L, O, R)**. **(A-C)** Prox1 single channel. **(D-F)** Peripherin single channel. **(G-I)** Prox1 and Peripherin double channels. **(J-L)** Prox1 and TuJ1 double channels. **(M-O)** Peripherin and TuJ1 double channels. **(P-R)** Prox1, Peripherin and TuJ1 triple channels. Prox1 expressing cells were positive for TuJ1, but negative for Peripherin throughout the cochlear turn. Peripherin positive cells were negative for Prox1 and weakly positive for TuJ1 (indicated by blue arrows). Scale bar: 50 μm. A cochlear cross section of postnatal SG (WT) at P14 immunostained against Prox1 (green), Peripherin (red) and NF200 (white). Each column represents different tonotopic positions: the basal turn **(A’, D’, G’, J’, M’, P’)**, the middle turn **(B’, E’, H’, K’, N’, Q’)** and the apical turn **(C’, F’, I’, L’, O’, R’)**. **(A’-C’)** Prox1 single channel. **(D’-F’)** Peripherin single channel. **(G’-I’)** Prox1 and Peripherin double channels. **(J’-L’)** Prox1 and NF200 double channels. **(M’-O’)** Peripherin and NF200 double channels. **(P’-R’)** Prox1, Peripherin and NF200 triple channels. Prox1 expressing cells were positive for NF200 throughout the cochlear turn. Peripherin positive cells were strongly positive for NF200 at the basal and middle turn (indicated by blue arrows). There was one Prox1 negative cell that expressed NF200 in the apex (indicated by yellow arrowheads). Scale bar: 50 μm.
(TIF)
