# Supplementary figures and images for: Dynamic Expression of Sox2, Gata3, and Prox1 during Primary Auditory Neuron Development in the Mammalian Cochlea
Source: PLoS One. 2017 Jan 24;12(1):e0170568. doi: 10.1371/journal.pone.0170568 (PMC5261741; doi:10.1371/journal.pone.0170568)

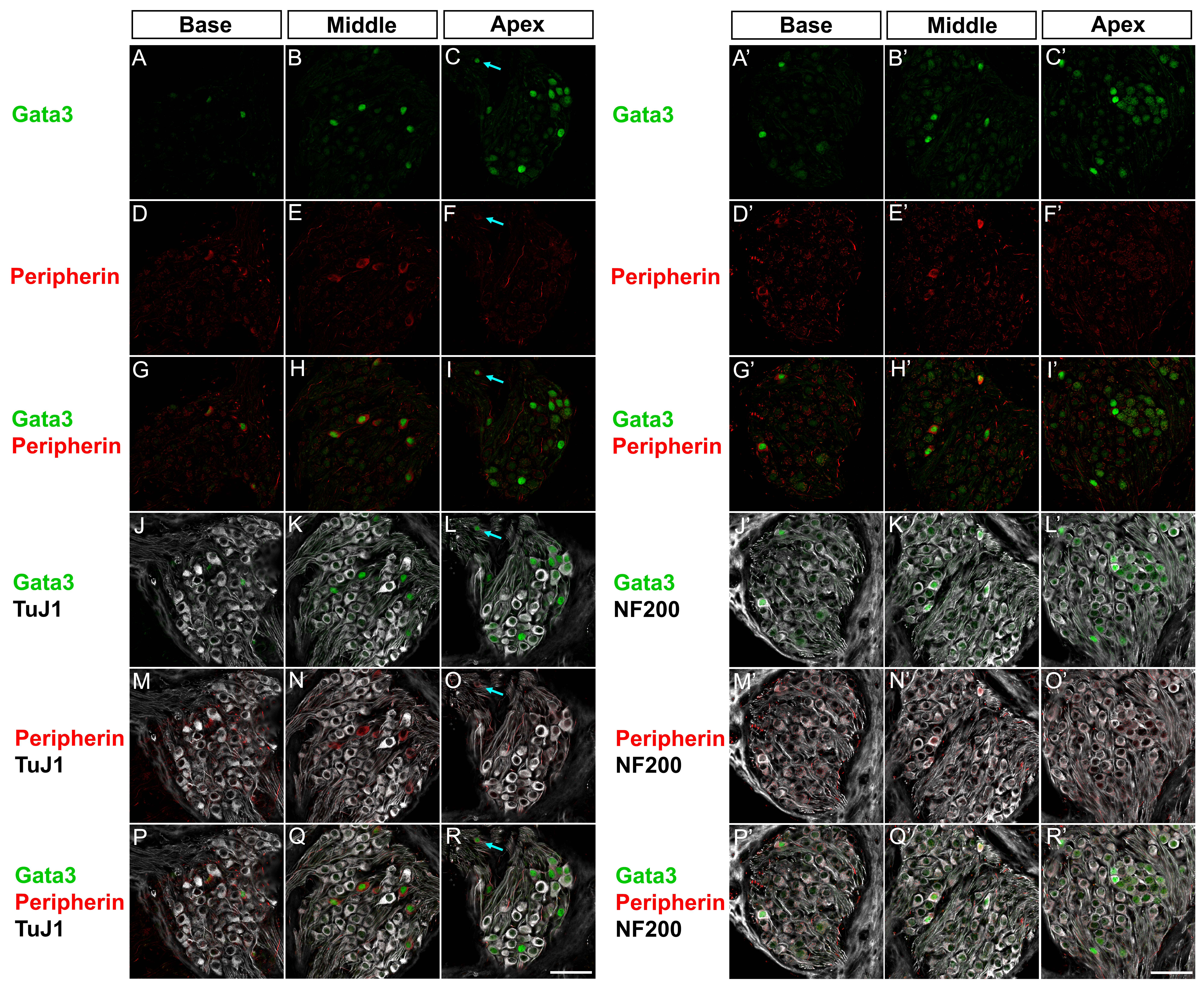

Supplement: S1 Fig — A cochlear cross section of postnatal SG (WT) at P14 immunostained against Gata3 (green), Peripherin (red) and TuJ1 (white). Each column represents different tonotopic positions: the basal turn (A, D, G, J, M, P), the middle turn (B, E, H, K, N, Q) and the apical turn (C, F, I, L, O, R). (A-C) Gata3 single channel. (D-F) Peripherin single channel. (G-I) Gata3 and Peripherin double channels. (J-L) Gata3 and TuJ1 double channels. (M-O) Peripherin and TuJ1 double channels. (P-R) Gata3, Peripherin and TuJ1 triple channels. Gata3 expressing cells were positive for Peripherin, but negative for TuJ1 at the basal and middle turn. There was one Peripherin positive cell in the apex, which was negative for TuJ1 (indicated by arrows in C, F, I, L, O, R). Scale bar: 50 μm. A cochlear cross section of postnatal SG (WT) at P14 immunostained against Gata3 (green), Peripherin (red) and NF200 (white). Each column represents different tonotopic positions: the basal turn (A’, D’, G’, J’, M’, P’), the middle turn (B’, E’, H’, K’, N’, Q’) and the apical turn (C’, F’, I’, L’, O’, R’). (A’-C’) Gata3 single channel. (D’-F’) Peripherin single channel. (G’-I’) Gata3 and Peripherin double channels. (J’-L’) Gata3 and NF200 double channels. (M’-O’) Peripherin and NF200 double channels. (P’-R’) Gata3, Peripherin and NF200 triple channels. Gata3 expressing cells were positive for Peripherin and strongly positive for NF200 at the basal and middle turn. There were no Peripherin positive cells in the apex. Scale bar: 50 μm. (TIF) [file pone.0170568.s001.tif]

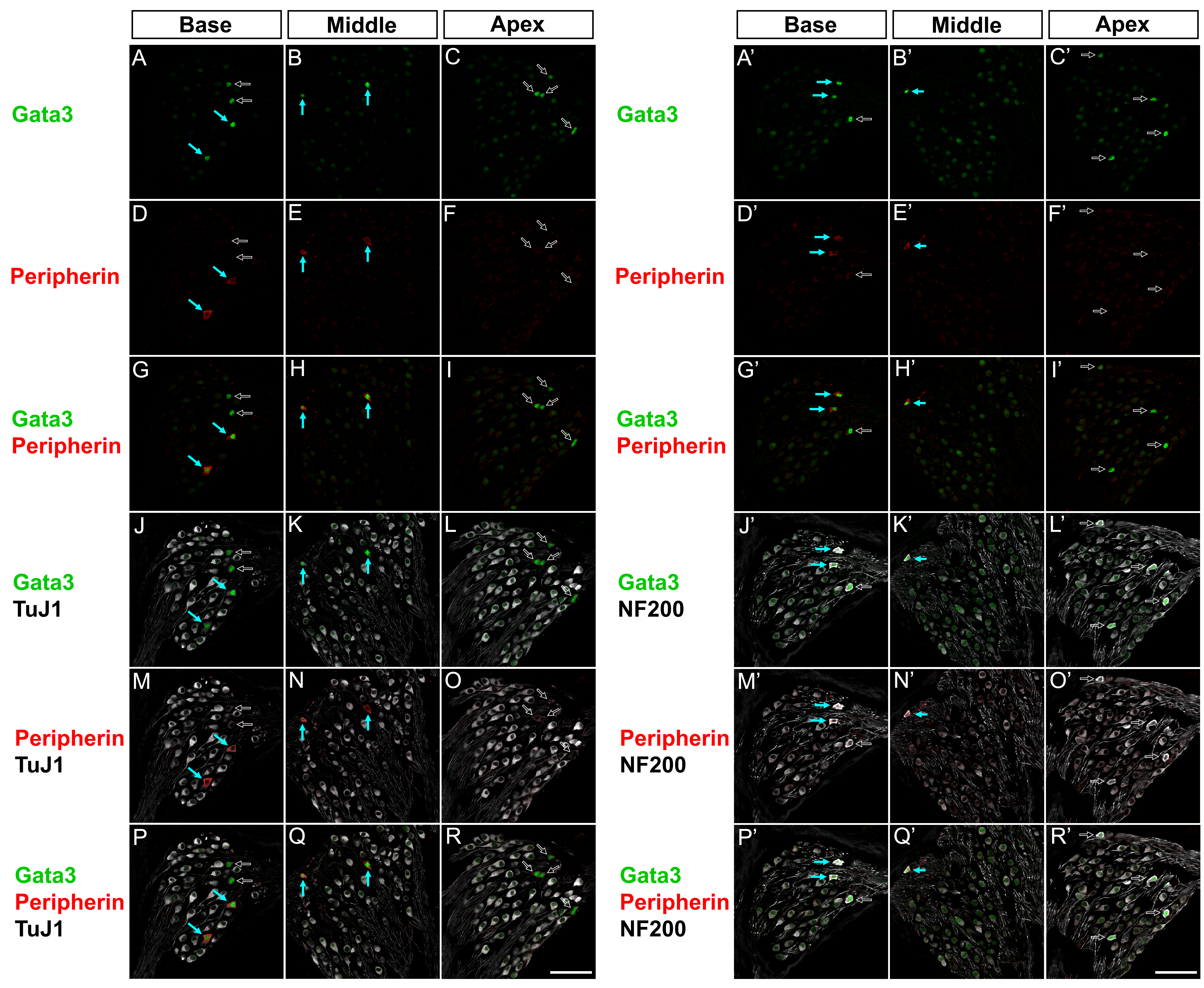

Supplement: S2 Fig — A cochlear cross section of postnatal SG (WT) at P35 immunostained against Gata3 (green), Peripherin (red) and TuJ1 (white). Each column represents different tonotopic positions: the basal turn (A, D, G, J, M, P), the middle turn (B, E, H, K, N, Q) and the apical turn (C, F, I, L, O, R). (A-C) Gata3 single channel. (D-F) Peripherin single channel. (G-I) Gata3 and Peripherin double channels. (J-L) Gata3 and TuJ1 double channels. (M-O) Peripherin and TuJ1 double channels. (P-R) Gata3, Peripherin and TuJ1 triple channels. Some Gata3 expressing cells were Peripherin positive, which were TuJ1 negative (indicated by blue arrows). Some Gata3 expressing cells were negative for Peripherin, which were TuJ1 negative (indicated by open arrows). There were no Peripherin positive cells in the apex. Scale bar: 50 μm. A cochlear cross section of postnatal SG (WT) at P35 immunostained against Gata3 (green), Peripherin (red) and NF200 (white). Each column represents different tonotopic positions: the basal turn (A’, D’, G’, J’, M’, P’), the middle turn (B’, E’, H’, K’, N’, Q’) and the apical turn (C’, F’, I’, L’, O’, R’). (A’-C’) Gata3 single channel. (D’-F’) Peripherin single channel. (G’-I’) Gata3 and Peripherin double channels. (J’-L’) Gata3 and NF200 double channels. (M’-O’) Peripherin and NF200 double channels. (P’-R’) Gata3, Peripherin and NF200 triple channels. Some Gata3 expressing cells were Peripherin positive, which were NF200 strongly positive (indicated by blue arrows). Some Gata3 expressing cells were negative for Peripherin, which were NF200 strongly positive (indicated by open arrows). There were no Peripherin positive cells in the apex. Scale bar: 50 μm. (TIF) [file pone.0170568.s002.tif]

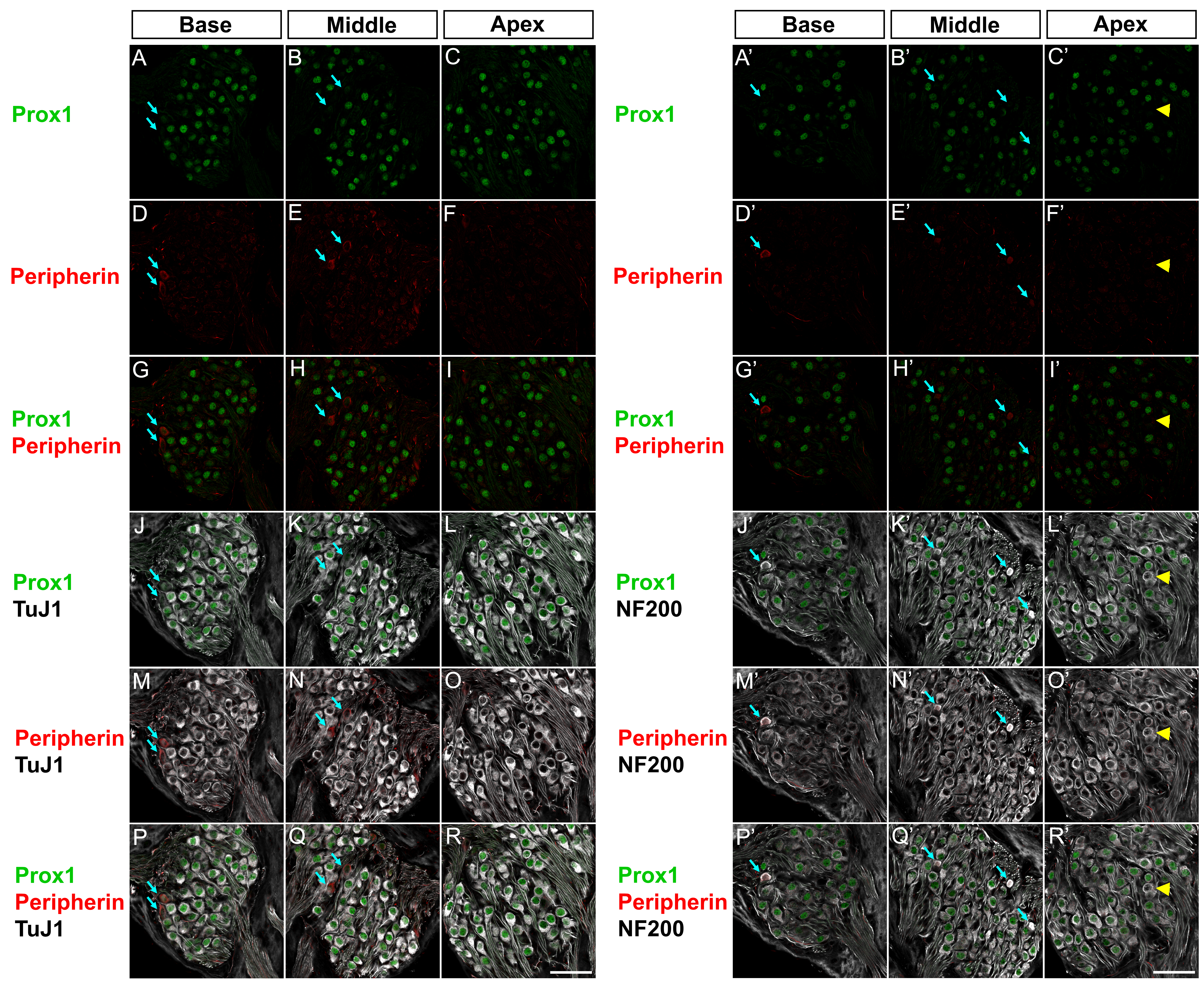

Supplement: S3 Fig — A cochlear cross section of postnatal SG (WT) at P14 immunostained against Prox1 (green), peripherin (red) and TuJ1 (white). Each column represents different tonotopic positions: the basal turn (A, D, G, J, M, P), the middle turn (B, E, H, K, N, Q) and the apical turn (C, F, I, L, O, R). (A-C) Prox1 single channel. (D-F) Peripherin single channel. (G-I) Prox1 and Peripherin double channels. (J-L) Prox1 and TuJ1 double channels. (M-O) Peripherin and TuJ1 double channels. (P-R) Prox1, Peripherin and TuJ1 triple channels. Prox1 expressing cells were positive for TuJ1, but negative for Peripherin throughout the cochlear turn. Peripherin positive cells were negative for Prox1 and weakly positive for TuJ1 (indicated by blue arrows). Scale bar: 50 μm. A cochlear cross section of postnatal SG (WT) at P14 immunostained against Prox1 (green), Peripherin (red) and NF200 (white). Each column represents different tonotopic positions: the basal turn (A’, D’, G’, J’, M’, P’), the middle turn (B’, E’, H’, K’, N’, Q’) and the apical turn (C’, F’, I’, L’, O’, R’). (A’-C’) Prox1 single channel. (D’-F’) Peripherin single channel. (G’-I’) Prox1 and Peripherin double channels. (J’-L’) Prox1 and NF200 double channels. (M’-O’) Peripherin and NF200 double channels. (P’-R’) Prox1, Peripherin and NF200 triple channels. Prox1 expressing cells were positive for NF200 throughout the cochlear turn. Peripherin positive cells were strongly positive for NF200 at the basal and middle turn (indicated by blue arrows). There was one Prox1 negative cell that expressed NF200 in the apex (indicated by yellow arrowheads). Scale bar: 50 μm. (TIF) [file pone.0170568.s003.tif]
